# N-terminal foamy virus Gag domains and individual residues are critical for capsid assembly and interaction with the unique Env leader protein Elp for Env-dependent budding

**DOI:** 10.1186/1742-4690-10-S1-P50

**Published:** 2013-09-19

**Authors:** Yang Liu, Martin Löchelt

**Affiliations:** 1 German Cancer Research Center DKFZ, Heidelberg, Germany

## Background

Foamy viruses (FVs) are distinct retroviruses with several features of their molecular biology and replication strategy clearly different from those of Orthoretroviruses like HIV, MLV and HTLV. One distinguishing feature of FVs is the absolute dependence of the cognate Env protein for Gag particle budding. Recent data showed that Gag has the capacity to bud from the cell, provided that a heterologous myristoylation signal is N-terminally appended. When this interaction is engineered to be reversible, even infectious particles are released. While the interaction surface of the FV-specific Env leader protein Elp required to specifically interact with Gag has been characterized to some degree, we set out to characterize the corresponding domain of Gag by separating the budding-relevant functions from those required for FV cytosolic Gag assembly.

## Materials and methods

Alanine scanning and single amino acid mutagenesis of the N-terminus of feline FV (FFV) Gag domain, together with cell biology and virological methods, were used to dissect FVparticle assembly and budding.

## Results

In addition to the centrally positioned arginine within the cytoplasmic targeting-retention signal (CTRS), other conserved residues upstream of the CTRS are involved in different phases of particle assembly, envelopment and budding. The different phenotypic changes of these mutants, including proteolytic Gag processing, intracellular Gag assembly into viral particles, and particle budding and infectivity, highlight their essential, distinct and only partially overlapping roles during viral assembly and budding. Importantly, there was a clear correlation between capsid formation and interaction with Elp, hinting at the requirement of higher order structures in Gag assemblies for Elp binding during budding. Finally, it appears that FV capsid assembly is a very efficient process, since monomeric Gag and lower molecular mass Gag aggregates were almost undetectable by the methods employed.

## Conclusion

The new insights into the spatial organization of the different functions within the FV Gag proteins will be presented and discussed with respect to the contribution of individual Gag residues for the different functions identified. The data implicate that FV assembly is a highly structured process and that components in this process are active only as specific molecular assemblies.

